# Liver‐Specific Suppression of PLA2G6/iPLA_2_β Improves Glucose and Lipid Metabolism in High‐Fat Diet‐Fed Mice

**DOI:** 10.1096/fj.202504753RR

**Published:** 2026-04-20

**Authors:** Kahori Shimizu, Mana Nishibata, Katsuyuki Nagata, Sho Hori, Shotaro Michinaga, Miyuki Kanehara, Miho Hashimoto‐Iwasaki, Fuminori Sakurai, Hideo Shindou, Koji Tomita, Toru Nishinaka, Hiroyuki Mizuguchi

**Affiliations:** ^1^ Laboratory of Biochemistry and Molecular Biology, Graduate School of Pharmaceutical Sciences The University of Osaka Osaka Japan; ^2^ Laboratory of Biochemistry and Molecular Biology, School of Pharmaceutical Sciences The University of Osaka Osaka Japan; ^3^ Laboratory of Biochemistry, Faculty of Pharmacy Osaka Ohtani University Osaka Japan; ^4^ Department of Molecular Biology The Jikei University School of Medicine Tokyo Japan; ^5^ Department of Pharmacodynamics Meiji Pharmaceutical University Tokyo Japan; ^6^ Laboratory of Biopharmaceutics, Faculty of Pharmacy Kindai University Osaka Japan; ^7^ Pharmaceutical Research and Technology Institute Kindai University Osaka Japan; ^8^ Department of Lipid Life Science, National Institute of Global Health and Medicine Japan Institute for Health Security Tokyo Japan; ^9^ Department of Medical Lipid Science, Graduate School of Medicine The University of Tokyo Tokyo Japan; ^10^ Laboratory of Molecular Biology, Faculty of Pharmacy Osaka Ohtani University Osaka Japan; ^11^ Global Center for Medical Engineering and Informatics The University of Osaka Osaka Japan; ^12^ Integrated Frontier Research for Medical Science Division, Institute for Open and Transdisciplinary Research Initiatives The University of Osaka Osaka Japan; ^13^ Center for Infectious Disease Education and Research The University of Osaka Osaka Japan

**Keywords:** adenovirus vector, diabetes mellitus, metabolic disorders, metabolic dysfunction‐associated steatotic liver disease, phospholipids, PLA2G6

## Abstract

Phospholipase A2 group VI (PLA2G6, also called iPLA_2_β) has been implicated in male fertility, neuronal disorders, and metabolic diseases. However, its therapeutic effects on metabolic disorders remain elusive. We investigated the effects of PLA2G6 suppression on glucose and lipid metabolism. Systemic inhibition of PLA2G6 in high‐fat diet‐fed mice reduced hepatic lipid droplet size without altering the levels of serum triglycerides and fasting blood glucose. Suppression of liver‐specific *Pla2g6* utilizing the short‐hairpin RNA knockdown technique using an adenovirus vector (Ad‐shPLA2G6) altered phospholipid and fatty acid metabolites and suppressed hepatic lipid accumulation, serum triglyceride, fasting glucose, and insulin levels. Additionally, Ad‐shPLA2G6 treatment downregulated lipid biosynthesis‐related genes but upregulated peroxisomal fatty acid oxidation‐related genes. These findings indicate that targeting hepatic *Pla2g6* modulates phospholipid and fatty acid metabolites and improves glucose and lipid metabolism, suggesting *Pla2g6* as a potential therapeutic target in metabolic disorders, including type 2 diabetes mellitus and metabolic dysfunction‐associated steatotic liver disease.

## Graphical abstract

Liver‐specific suppression of PLA2G6/iPLA2β by utilizing the short‐hairpin RNA knockdown technique using an adenovirus vector (Ad‐shPLA2G6) altered phospholipid and fatty acid metabolites and suppressed hepatic lipid accumulation, serum triglyceride, and fasting glucose levels.

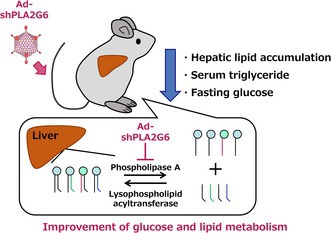

## 1 Introduction

The global incidence of metabolic disorders such as type 2 diabetes mellitus (T2DM) and metabolic dysfunction‐associated steatotic liver disease (MASLD) has increased markedly in recent years. T2DM accounts for approximately 90% of all cases of diabetes mellitus and is characterized by insulin resistance, impaired insulin secretion from pancreatic β cells, or both. These defects associated with T2DM reduce glucose uptake in peripheral tissues, leading to hyperglycemia [1]. Insulin resistance refers to an insufficient cellular response to normal physiological levels of insulin across different tissues and is a key contributor to liver metabolic diseases, such as MASLD (formerly known as nonalcoholic fatty liver disease). MASLD is characterized by an excessive accumulation of intrahepatic triglycerides (TGs) [2, 3] and is one of the most common chronic hepatic diseases. MASLD covers a wide spectrum of liver diseases, including hepatosteatosis, liver fibrosis, cirrhosis, and hepatocellular carcinoma [4, 5, 6]. Clinical data indicate that MASLD affects approximately 55.5% of patients with T2DM [7] and is an independent risk factor for T2DM, suggesting a strong association between the two conditions.

Phospholipid metabolism is also involved in the pathogenesis of metabolic disorders. Phospholipids, the major components of cellular membranes, consist of two fatty acids and one polar head group linked to a glycerol backbone. Phospholipase A_2_ (PLA_2_) catalyzes the hydrolysis of the *sn*‐2 position of phospholipids to generate free fatty acids (FFAs), including arachidonic acid and lysophospholipids. Arachidonic acid is then metabolized by cyclooxygenase, lipoxygenase, and cytochrome P450 enzymes into eicosanoids such as prostaglandin E_2_ (PGE_2_). Lysophospholipids are remodeled back to phospholipids by lysophospholipid acyltransferases (LPLATs) [8, 9, 10]. The metabolism of phospholipids has been associated with the development of metabolic disorders such as T2DM and MASLD. For example, mice with targeted inactivation of the group 1B phospholipase A2 (*Pla2g1b*) gene exhibit lower postprandial glycemia than wild‐type mice [11]. Depletion of LPLAT11, also known as lysophosphatidylinositol acyltransferase 1 and membrane‐bound *O*‐acyltransferase domain‐containing 7, in adult mice causes hepatic steatosis [12]. Furthermore, overexpression of liver‐specific LPLAT10 (also called lysophosphatidylethanolamine acyltransferase 2 and lysophosphatidylcholine acyltransferase 4) [10, 13, 14] increases glucose‐stimulated insulin secretion [15]. Together, these findings suggest that regulating phospholipid metabolism may help treat metabolic disorders.

PLA2G6, also known as group VIA PLA_2_ and iPLA_2_β, belongs to the patatin‐like phospholipase domain‐containing protein (PNPLA) family. PLA2G6/iPLA_2_β/PNPLA9, a prototypical iPLA_2_, plays a housekeeping role in phospholipid metabolism [16] as well as crucial roles in male fertility [17], neuronal disorders [18], and metabolic diseases [19, 20, 21, 22]. Previous reports have demonstrated that PLA2G6 does not exhibit exclusive specificity for arachidonic acid [23] and preferentially acts on phospholipids with palmitic acid, thus providing the corresponding palmitoyl lysophospholipid [20, 21]. Deng et al. observed that *Pla2g6* knockout ob/ob mice, genetically deficient in leptin, exhibited attenuation of hepatic steatosis through hepatic phospholipid remodeling. However, the therapeutic effects of PLA2G6 on metabolic disorders remain elusive [20].

Here, we hypothesized that the suppression of *Pla2g6* may reduce hepatic lipid accumulation, thereby alleviating MASLD and improving insulin sensitivity. We aimed to determine whether PLA2G6 suppression could ameliorate glucose and lipid metabolic dysfunction. First, we used FKGK18, an inhibitor of PLA2G6, to systemically suppress PLA2G6 in mice. Next, to suppress liver‐specific PLA2G6, we used a short‐hairpin RNA knockdown system using an adenovirus (Ad) vector, as systemic administration of Ad vectors results in liver‐specific expression of exogenous genes [24]. Among the Ad vectors, we employed an improved Ad vector, referred to as Ad‐E4‐122aT [25, 26], which demonstrated superior and more sustained transgene expression along with reduced hepatotoxicity compared to that of conventional Ad vectors. The findings of the present study demonstrate the effect of liver‐specific suppression of PLA2G6 on glucose and lipid metabolism, positioning PLA2G6 as a novel target for the treatment of MASLD and T2DM.

## 2 Methods

### 2.1 Sex as a Biological Variable

Our study exclusively examined male mice. This design was chosen based on the knowledge that the prevalence of T2DM and MASLD is higher in males, making male mice a relevant model for these conditions.

### 2.2 Mice and Cells

Six‐week‐old male C57BL/6N mice were purchased from Nippon SLC (Hamamatsu, Japan). A high‐fat diet (HFD; High Fat Diet 32, 56.7% kcal from fat) was obtained from CLEA Japan (Tokyo, Japan). The PLA2G6 inhibitor FKGK18 (20 mg/kg body weight, Cayman Chemical, MI, USA) or DMSO (control) was administered intraperitoneally every 3 days, concurrently with HFD feeding. The FKGK18 dose was selected based on a previous report [27]. Ad vectors were intravenously administered to mice at 5 × 10^9^ infectious units (IFU) per mouse via the tail vein and were simultaneously fed an HFD. Mice were maintained on a 12/12‐h light/dark cycle with *ad libitum* access to water and food. At the experimental endpoint, the mice were anesthetized with 3% isoflurane and dissected. No relevant adverse effects were observed in the experimental animals. All efforts were made to minimize animal suffering. HEK293 cells were cultured in Dulbecco's modified Eagle's medium (FUJIFILM Wako Pure Chemical Corporation, Osaka, Japan) supplemented with 10% FBS, 2 mmol/L glutamine (Nacalai Tesque, Kyoto, Japan), and antibiotics. Primary mouse hepatocytes and nonparenchymal cells were isolated from normal diet (ND)‐ or HFD‐fed C57BL/6 mice using the hepatic portal perfusion technique, as described previously [28, 29].

### 2.3 Plasmids and Ad Vectors

Ad vectors were constructed using an improved in vitro ligation method as described previously [30, 31]. The shuttle plasmid pHM5‐hU6/hH1 was designed to express shRNA by inserting the desired sequence into the *Swa*I/*Sal*I site [32]. To generate shRNA targeting *Pla2g6*, oligonucleotides (Table S1) were synthesized, annealed, and cloned into the *Swa*I/*Sal*I sites downstream of the hU6 promoter, producing pHM5‐hU6/hH1‐shPLA2G6. An shRNA targeting firefly luciferase was constructed using pHM5‐hU6/hH1, producing pHM5‐hU6/hH1‐shLuc [32]. pHM5‐hU6/hH1‐shPLA2G6 and pHM5‐hU6/hH1‐shLuc were digested with I‐*Ceu*I/PI‐*Sce*I, and the resulting fragments were ligated into the corresponding I‐CeuI/PI‐SceI‐digested Ad vector plasmid, pAdHM4‐E4‐122aT [25, 26], producing pAd‐shPLA2G6 and pAd‐shLuc, respectively. Each Ad vector plasmid was then digested with *Pac*I to release the recombinant viral genome and transfected into HEK293 cells cultured in 60‐mm dishes using Lipofectamine 3000 (Thermo Fisher Scientific, Waltham, MA, USA). Ad‐shPLA2G6 and Ad‐shLuc vectors were propagated in HEK293 cells and purified, as described previously [25]. Virus particle (VP) counts were determined spectrophotometrically [33], and biological titers were measured using an Adeno‐X‐rapid titer kit (Clontech, Mountain View, CA, USA). The particle‐to‐biological titer ratio for all Ad vectors used in this study ranged between 5.1 and 7.3.

### 2.4 Hepatotoxicity Analysis

Blood samples were collected from mice via retro‐orbital bleeding, and serum was obtained by centrifugation. Serum alanine aminotransferase (ALT) and aspartate aminotransferase (AST) levels were determined using a transaminase‐CII kit (FUJIFILM Wako Pure Chemical Corporation).

### 2.5 Histological Analysis

For histopathological examination of mouse tissues, tissue samples were collected, washed with PBS, and fixed in 10% buffered formalin or 4% paraformaldehyde. The samples were embedded in paraffin, sectioned (2–3‐μm thick), and stained with hematoxylin and eosin at Applied Medical Research (Osaka, Japan). Quantitative morphometric analysis of hepatic lipid droplets was performed using ImageJ (NIH, Bethesda, MD, USA). Vacuoles consistent with lipid droplets were identified from hematoxylin and eosin‐stained liver sections by intensity thresholding, followed by binarization and particle analysis. The area of individual lipid droplet–associated vacuoles was measured for each field. Large vascular structures were excluded based on size filtering. The median lipid droplet area was calculated from the analyzed fields and used for statistical comparison. In addition, the frequency distribution of lipid droplet area was analyzed using pooled lipid droplets across multiple fields within each group and presented as a percentage of total droplets. For Oil Red O staining, the mouse tissue samples were washed with phosphate‐buffered saline and fixed in 4% paraformaldehyde. Frozen sections were prepared and stained with an Oil Red O staining solution at Applied Medical Research. For semi‐quantitative histopathological comparison, each section was analyzed using ImageJ.

### 2.6 Liver TG Levels

The liver tissue was homogenized, and lipids were extracted using the Folch method [34]. The extracted lipids were dried and reconstituted in 2‐propanol. TG levels in the liver were determined using the LabAssay Triglyceride Kit (FUJIFILM Wako Pure Chemical Corporation).

### 2.7 Glucose Tolerance Test

Glucose tolerance tests were performed on 16‐h‐fasted C57BL/6 mice intraperitoneally injected with 1 g/kg glucose. Blood glucose levels were determined immediately before and at the indicated times after injection using Glutest Sensor Neo (Sanwa Kagaku Kenkyusho, Nagoya, Japan).

### 2.8 Serum Insulin, TG, FFA, and Cholesterol Levels

Blood samples were collected from mice via retro‐orbital bleeding, and serum was obtained by centrifugation. Serum insulin, TG, and FFA levels in mice were determined using Ultrasensitive Mouse Insulin ELISA kits (Mercodia, Uppsala, Sweden), the LabAssay Triglyceride Kit (FUJIFILM Wako Pure Chemical Corporation), and LabAssay NEFA (FFA; FUJIFILM Wako Pure Chemical Corporation), respectively, according to the manufacturer's instructions. Serum total and high‐density lipoprotein (HDL) cholesterol levels were measured using a Fuji DRI‐CHEM automated analyzer (Fujifilm, Tokyo, Japan). The HOMA‐IR was calculated using the following equation:

$$\begin{array}{c} \text{HOMA‐}\mathrm{IR}=\text{fasting glucose}\;\left(\mathrm{mg}/\mathrm{dL}\right)\\ \;\times \text{fasting insulin}\;\left(\mathrm{mIU}/\mathrm{mL}\right)/405 \end{array}$$

### 2.9 Quantitative Reverse Transcription (RT)‐PCR Analysis of Gene Expression in Mouse Tissues

Total RNA was extracted from mouse tissues using TRIzol reagent (Thermo Fisher Scientific), according to the manufacturer's instructions. Total pancreatic RNA was isolated as described previously [35]. The mRNA levels were determined using quantitative RT‐PCR with THUNDERBIRD SYBR qPCR Mix (TOYOBO, Osaka, Japan). The reaction was carried out using the primers listed in Table S2 and following the thermal cycling protocol: 60 s at 95°C, followed by 40 cycles of 15 s at 95°C, and 60 s at 63°C. The mRNA level of the target genes was normalized to that of *Actb*.

### 2.10 RNA‐Seq Analysis

Total RNA was extracted from the livers using TRIzol reagent and purified using a Monarch Spin RNA Cleanup Kit (New England Biolabs, Ipswich, MA, USA). RNA concentration and quality were assessed using a NanoDrop spectrophotometer (Thermo Fisher Scientific) and a TapeStation (Agilent Technologies, Santa Clara, CA, USA). Libraries were prepared using the SMART‐Seq v4 Ultra Low Input RNA kit, and sequencing was performed by Takara Bio Inc. (Shiga, Japan) on a NovaSeq platform (Illumina, San Diego, CA, USA) to generate 150‐bp paired‐end reads. Raw sequence data were processed for quality control, read trimming, and mapping using the proprietary pipeline of Takara Bio. Reads were aligned to the reference genome, mouse GPCm39, and gene expression levels were quantified as transcripts per million (TPM). Differential gene expression analysis was performed using Welch's *t*‐test, which does not assume equal variance between the groups.

### 2.11 Gene Ontology (GO) Enrichment Analysis

GO enrichment analysis was performed using the Database for Annotation, Visualization, and Integrated Discovery (DAVID) [36, 37]. Differentially expressed genes were submitted to DAVID for functional annotation, and the enrichment of GO terms (biological processes) was assessed in March 2024. Statistical significance was evaluated using a modified Fisher's exact test (EASE score), and GO terms with a *p*‐value of < 0.05 were considered significantly enriched.

### 2.12 Phospholipid Quantification Using Liquid Chromatography–Tandem Mass Spectrometry (LC–MS/MS)

Frozen liver tissue was homogenized in saline, and lipids were extracted using the Bligh and Dyer method [38]. The extracted lipids were dried using a centrifugal evaporator and reconstituted in methanol. LC–MS/MS was performed using a Nexera Ultra High‐Performance Liquid Chromatography (UHPLC) system and triple quadrupole mass spectrometer (LC–MS‐8050; Shimadzu Corp., Japan). Lipid samples (5 μL/injection) were separated on an Acquity UPLC BEH C8 column (1.7 μm, 2.1 × 100 mm, Waters) at a flow rate of 0.35 mL/min with a gradient of mobile phase A (5 mM NH_4_HCO_2_ in water), B (acetonitrile), and C (isopropyl alcohol). The column oven temperature was set to 47°C. The gradient setting was as follows: time (min), (%A/%B/%C): 0 (50/45/5), 10 (20/75/5), 20 (20/50/30), 27.5–28.5 (5/5/90), and 28.6 (50/45/5). Phosphatidylcholine (PC), lysophosphatidylcholine (LPC), and phosphatidylethanolamine (PE) species were detected in positive ion mode following multiple reaction monitoring (MRM) transitions, with (Q1, Q3): ([M + H]^+^, 184) for PC and LPC; (Q1, Q3), ([M + H]^+^, neutral loss of 141) for PE. Data were analyzed using TRACES [39].

### 2.13 Fatty Acid Metabolite Measurement Using LC–MS

Frozen liver tissue was pulverized using an AUTOMILL (Tokken, Chiba, Japan), and lipids were extracted with methanol for 60 min at 4°C in the presence of a deuterium‐labeled internal standard mixture. Lipid extracts were purified by solid‐phase extraction using an Oasis HLB column (Waters). Fatty acid metabolites were measured using a Nexera UHPLC system and a triple quadrupole mass spectrometer LCMS‐8060 (Shimadzu Corp., Japan) as previously described [40] with slight modifications. Briefly, the lipid samples (5 μL/injection) were separated on a Kinetex C8 column (2.6 μm, 2.1 × 150 mm, Phenomenex) at a flow rate of 0.4 mL/min with a gradient of mobile phase A (0.1% formic acid in water), B (acetonitrile), and C (isopropyl alcohol). The column oven temperature was set to 40°C. The gradient setting was as follows: time (min) (%A/%B/%C): 0 (90/10/0), 5 (75/25/0), 10 (65/35/0), 20 (25/75/0), 20.1–27 (5/95/0), 30–35 (5/50/45), 38–42 (5/5/90), 42.1–44 (5/95/0), and 44.1–48 (90/10/0). Raw data were analyzed using LabSolutions Insight (Shimadzu Corp., Japan).

### 2.14 Statistical Analyses

Statistical analyses were performed using BellCurve for Excel (Social Survey Research Information Co. Ltd., Tokyo, Japan). The Mann–Whitney *U* test was used to compare the differences between two independent groups, and one‐way ANOVA with Dunnett's post hoc test or Tukey–Kramer test was used for multiple comparisons. Data are presented as the mean ± standard error, and differences with a *p*‐value of < 0.05 were considered statistically significant.

## 3 Results

### 3.1 PLA2G6 Inhibitor Treatment Reduced Hepatic Lipid Accumulation

**FIGURE 1:**
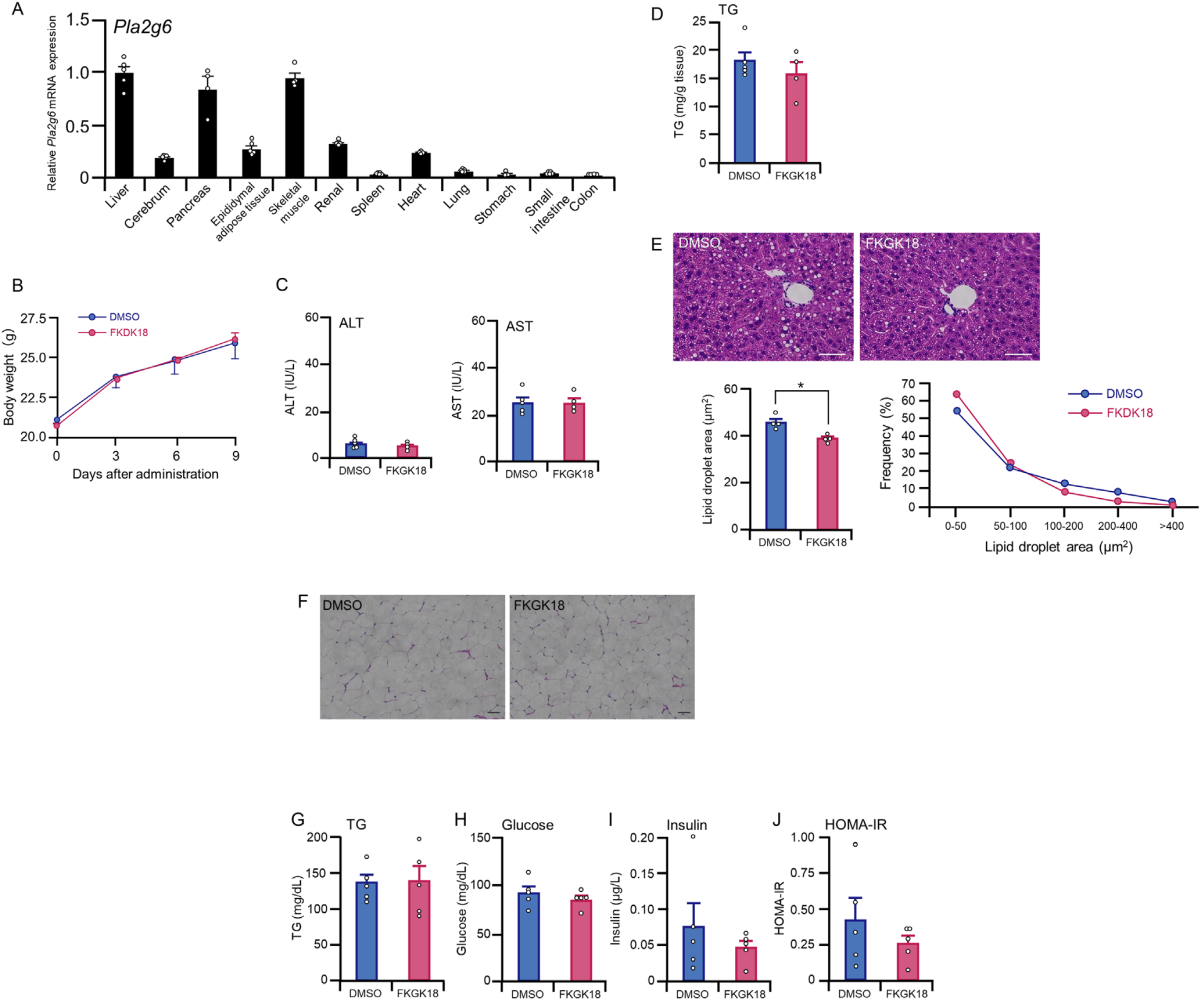
Tissue distribution of Pla2g6 and effects of PLA2G6 inhibition in high‐fat diet (HFD)‐fed C57BL/6 mice on glucose and lipid metabolism. (A) Relative expression of *Pla2g6* mRNA levels in different tissues of normal diet (ND)‐fed male C57BL/6 mice measured using quantitative RT‐PCR. *Pla2g6* mRNA levels in the liver were set to 1.0. (B–J) HFD‐fed C57BL/6 male mice were intraperitoneally administered with FKGK18 (PLA2G6 inhibitor) at 20 mg/kg body weight or DMSO every 3 days for 10 days. (B) Body weight changes in FKGK18‐ and DMSO‐treated HFD‐fed C57BL/6 mice. (C) Serum ALT and AST levels; (D) hepatic TG levels; (E) hematoxylin and eosin staining of the liver, median lipid droplet area in the liver, and frequency distribution of lipid droplet area in the liver; (F) hematoxylin and eosin staining of epididymal adipose tissue; (G) fasting serum TG; (H) blood glucose; (I) serum insulin; and (J) HOMA‐IR levels in HFD‐fed C57BL/6 mice 10 days after the initiation of FKGK18 or DMSO treatment. Scale bar in D and E = 50 μm. Data are expressed as mean ± SE (*n* = 4–5). Differences between the groups were analyzed using the Mann–Whitney *U* test. *p < 0.05.

To examine the tissue distribution of PLA2G6, *Pla2g6* mRNA levels were determined in different tissues of ND‐fed male C57BL/6 mice. *Pla2g6* mRNA was detected in all tissues, with pronounced expression levels in the liver, pancreas, and skeletal muscle tissues (Figure 1A). Subsequently, to investigate the effect of systemic suppression of *Pla2g6* on glucose and lipid metabolism, C57BL/6 mice were fed an HFD and administered either dimethyl sulfoxide (DMSO) or FKGK18 (a PLA2G6 inhibitor; 20 mg/kg body weight) intraperitoneally every 3 days. Assessment of the effects of FKGK18 treatment on body weight revealed no significant differences between the groups on different days after the treatment (Figure 1B). Similarly, FKGK18 administration did not alter the serum ALT and AST levels, markers of hepatotoxicity, compared with levels in response to DMSO administration at 10 days after treatment initiation, indicating the safety of FKGK18 (Figure 1C). Hepatic TG content revealed no significant difference between FKGK‐ and DMSO‐treated mouse groups; however, histopathological analysis of hematoxylin–eosin‐stained liver sections demonstrated a marked shift in lipid droplet size distribution (Figure 1D,E). In particular, the median lipid droplet size was significantly smaller in the FKGK‐treated mouse group, indicating altered hepatic lipid storage patterns without changes in total TG burden. Histopathological analyses of the epididymal adipose tissue sections indicated smaller adipocyte deposition in the epididymal adipose tissue of FKGK18‐treated mice compared with that in DMSO‐treated mice (Figure 1F). No significant differences were observed in serum TG (Figure 1G) and fasting glucose (Figure 1H) levels between FKGK18‐ and DMSO‐treated mice. Fasting insulin levels (Figure 1I) and homeostasis model assessment of insulin resistance (HOMA‐IR), an index of insulin resistance (Figure 1J), tended to be lower in FKGK18‐treated mice than in DMSO‐treated mice. These findings indicate that *Pla2g6* inhibition via FKGK18 treatment reduced hepatic lipid droplet size without altering serum TG levels and glucose metabolism.

### 3.2 Liver‐Specific PLA2G6 Suppression Alters Phospholipid Composition and Fatty Acid Metabolites in HFD‐Fed Mice

**FIGURE 2:**
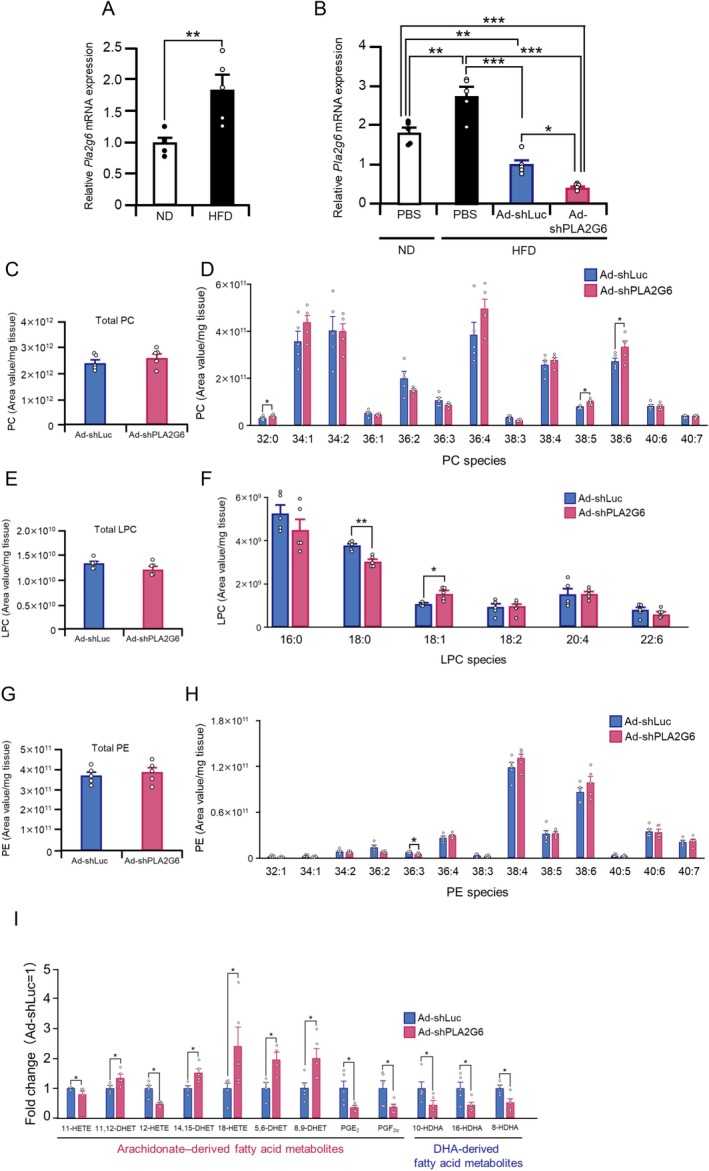
Hepatic *Pla2g6* mRNA expression and lipid profiles in mice exhibiting liver‐specific *Pla2g6* suppression. (A) Hepatic *Pla2g6* expression in C57BL/6 mice fed a normal diet (ND) or high‐fat diet (HFD) for 4 weeks, starting at 6 weeks of age. Values in ND‐fed mice were set to 1.0. (B–I) C57BL/6 mice on HFD received intravenous injections (tail vein) of 5 × 10^9^ infectious units (IFU)/mouse of adenovirus (Ad) vectors expressing short hairpin RNA (shRNA) targeting PLA2G6 (Ad‐shPLA2G6) or control luciferase shRNA (Ad‐shLuc). (B) Hepatic *Pla2g6* mRNA levels at 10 days after the administration of Ad‐shPLA2G6, Ad‐shLuc, or PBS. *Pla2g6* mRNA levels in Ad‐shLuc‐treated mice were set to 1.0. (C–H) Levels of total PC (C), PC species (D), total LPC (E), LPC species (F), total PE (G), and PE species (H) in the livers of HFD‐fed C57BL/6 mice 10 days after administration of Ad‐shPLA2G6 or Ad‐shLuc. (I) Fatty acid metabolites with significant differences in the liver of HFD‐fed C57BL/6 mice 10 days after treatment with Ad‐shPLA2G6 compared with Ad‐shLuc. The Mann–Whitney *U* test was used to compare the differences between two independent groups (A, C–I). One‐way ANOVA with Tukey–Kramer test was used for multiple comparisons (B). The data are expressed as mean ± SE values (*n* = 4–5). ND, normal diet; HFD, high‐fat diet. *p < 0.05, **p < 0.01, ***p < 0.001.

Assessment of PLA2G6 levels in the liver in HFD‐fed or ND‐fed C57BL/6 mice indicated a 1.8‐fold higher expression of *Pla2g6* mRNA in HFD‐fed mice than in ND‐fed mice (Figure 2A). *Pla2g6* mRNA levels in the hepatocytes isolated from HFD‐fed mice were higher than those in the hepatocytes isolated from ND‐fed mice (Figure S1A). *Pla2g6* mRNA levels were similar between the HFD‐ and ND‐fed mouse nonparenchymal cells (Figure S1B). These results suggest that *Pla2g6* mRNA level upregulation occurs within hepatocytes in HFD‐fed mice.

As *Pla2g6* inhibition reduced hepatic lipid accumulation in HFD‐fed mice, we hypothesized that the liver‐specific suppression of PLA2G6 would improve glucose and lipid metabolism. To test this hypothesis, *Pla2g6* was specifically suppressed in the mouse liver using short hairpin RNA (shRNA) knockdown technology using an Ad vector expressing *Pla2g6* shRNA (Ad‐shPLA2G6). HFD‐fed C57BL/6 mice were subjected to intravenous injections of Ad‐shPLA2G6 or control luciferase shRNA vector (Ad‐shLuc), and the effects were assessed 10 days later. Hepatic *Pla2g6* mRNA levels were significantly increased in PBS‐treated HFD‐fed mice compared with PBS‐treated ND‐fed mice (Figure 2B). The reduction in hepatic *Pla2g6* expression observed in Ad‐shLuc‐treated HFD‐fed mice compared with PBS‐treated HFD‐fed mice was transient, as *Pla2g6* levels were comparable between the two groups at 8 weeks after Ad‐shLuc or PBS administration (Figure S2). Hepatic *Pla2g6* levels were reduced to a greater extent in Ad‐shPLA2G6‐treated HFD‐fed mice than in Ad‐shLuc‐treated HFD‐fed mice, with a 2.5‐fold greater reduction (Figure 2B). Furthermore, *Pla2g6* mRNA levels in the epididymal adipose tissue were comparable among the Ad‐shPLA2G6‐, Ad‐shLuc‐, and PBS‐treated HFD‐fed mice (Figure S3), suggesting liver‐specific inhibition of *Pla2g6* expression.

Next, we performed lipidomics analysis using LC–MS/MS to assess the effects of *Pla2g6* suppression on phospholipid composition. The results revealed no differences in total hepatic PC and LPC between Ad‐shPLA2G6‐ and Ad‐shLuc‐treated mice (Figure 2C,E). However, significant alterations were observed in the PC and LPC subspecies. For instance, the levels of PC 32:0, PC 38:5, PC 38:6, and LPC 18:1 were significantly higher, whereas those of LPC 18:0 were significantly lower in the livers of Ad‐shPLA2G6‐treated mice than in those of Ad‐shLuc‐treated mice (Figure 2D,F). Furthermore, total hepatic PE did not differ between the groups (Figure 2G), whereas PE 36:3 was significantly reduced in Ad‐shPLA2G6‐treated mice (Figure 2H).

Quantification of the fatty acid metabolites in the liver using LC–MS/MS demonstrated that among the 51 fatty acid metabolites examined in this study, 12 differed significantly between Ad‐shPLA2G6‐ and Ad‐shLuc‐treated mice (Figure 2I and Table S3). Among arachidonic‐derived fatty acid metabolites, the amounts of PGE_2_ and PGF_2α_ were 2.6‐fold lower in the livers of Ad‐shPLA2G6‐treated mice than in those of Ad‐shLuc‐treated mice. The levels of 11‐HETE and 12‐HETE were also reduced in Ad‐shPLA2G6‐treated mice. In contrast, the levels of hepatic 11,12‐DHET, 14,15‐DHET, 18‐HETE, 5,6‐DHET, and 8,9‐DHET were significantly higher in Ad‐shPLA2G6‐treated mice than in Ad‐shLuc‐treated mice. Among docosahexaenoate (DHA)‐derived fatty acid metabolites, 10‐HDHA, 16‐HDHA, and 8‐HDHA levels were 1.9‐ to 2.9‐fold lower in the livers of Ad‐shPLA2G6‐treated mice than in those of Ad‐shLuc‐treated mice. These findings suggest that liver‐specific suppression of *Pla2g6* modifies distinct PC, LPC, and PE subspecies and alters fatty acid metabolite profiles in HFD‐fed mice.

### 3.3 Suppression of PLA2G6 Expression in the Liver Reduces Hepatic Lipid Accumulation and Serum TG Levels

**FIGURE 3:**
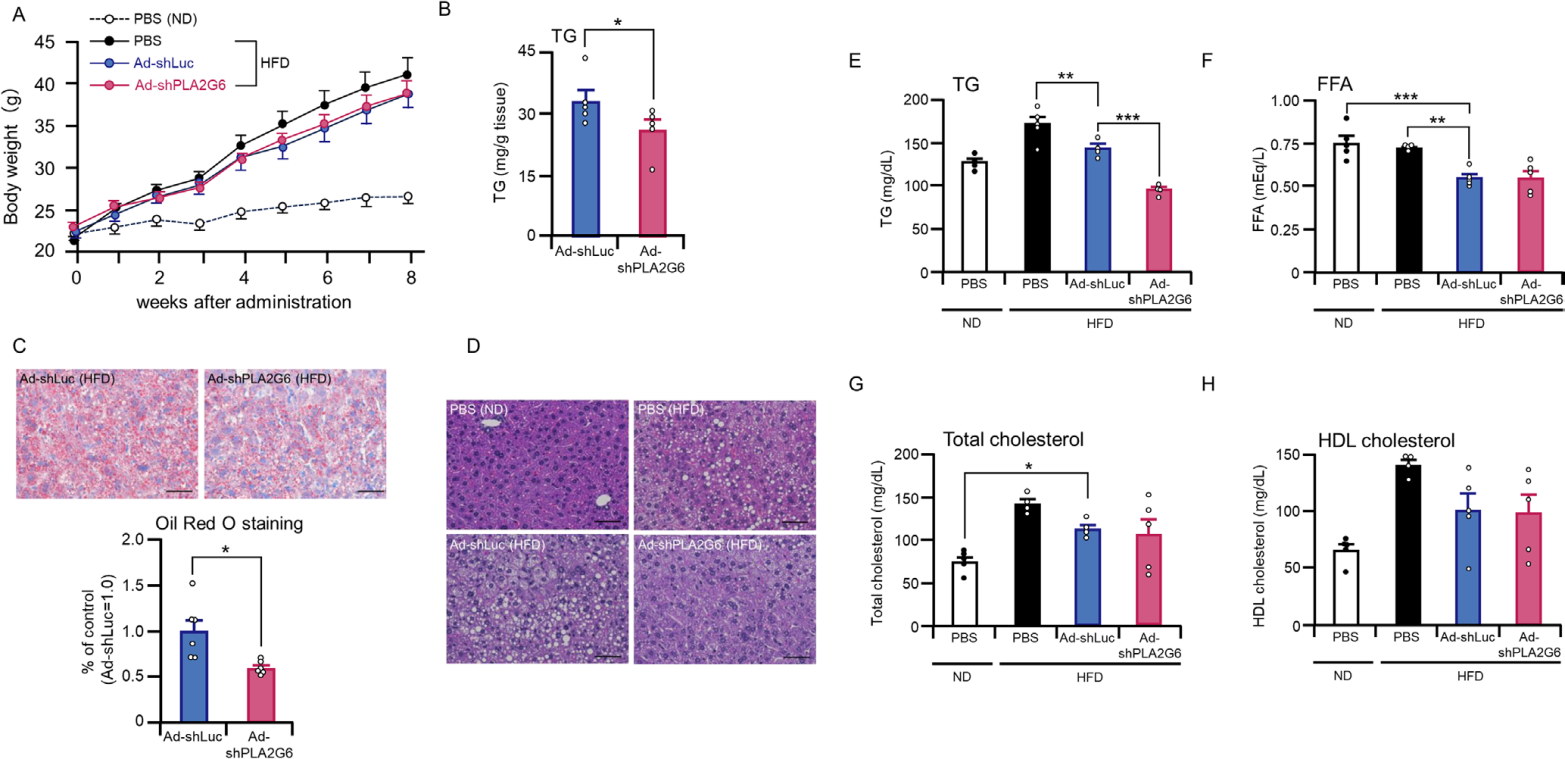
Suppression of *Pla2g6* expression in the liver reduces hepatic lipid accumulation and serum triglyceride levels. C57BL/6 mice on a high‐fat diet (HFD) received intravenous tail vein injections of 5 × 10^9^ IFU/mouse of Ad‐shPLA2G6, Ad‐shLuc, or PBS. (A) Body weight changes in normal diet (ND)‐ or HFD‐fed C57BL/6 mice. (B–D) (B) Hepatic TG levels, representative images of (C) Oil Red O or (D) hematoxylin and eosin‐stained liver sections from ND‐ or HFD‐fed C57BL/6 mice obtained 10 days after administration of Ad‐shPLA2G6, Ad‐shLuc, or PBS. Scale bar = 50 μm. Semi‐quantitative analysis of Oil red O staining performed using ImageJ. (E–H) Fasting serum (E) TG, (F) free fatty acid (FFA), (G) total cholesterol, and (H) HDL cholesterol levels in ND‐ or HFD‐fed C57BL/6 mice at 10 days after the administration of Ad‐shPLA2G6, Ad‐shLuc, or PBS. Data are expressed as mean ± SE (*n* = 4–5). *p < 0.05, **p < 0.01, ***p < 0.001, versus Ad‐shLuc treatment. One‐way ANOVA with Dunnett's post hoc test was used for multiple comparisons (A, C, and D).

To assess the metabolic effects of hepatic PLA2G6 suppression, the body weights of the mice were monitored for 8 weeks after Ad vector administration and HFD feeding. Body weight gain was comparable among Ad‐shPLA2G6‐, Ad‐shLuc‐, and PBS‐treated HFD‐fed mice (Figure 3A). Hepatic TG content at 10 days after administration of Ad vector treatment was significantly reduced in Ad‐shPLA2G6‐treated HED‐fed mice than in Ad‐shLuc‐treated HFD‐fed mice (Figure 3B). Consistent with this biochemical finding, Oil Red O staining of liver sections also revealed a lower amount of lipid droplets in Ad‐shPLA2G6‐treated HFD‐fed mice than in Ad‐shLuc‐treated HFD‐fed mice (Figure 3C). Representative hematoxylin–eosin staining further supported the reduction in accumulation of lipid droplets (Figure 3D). The size of adipocytes in the epididymal adipose tissue of Ad‐shLuc‐ and Ad‐shPLA2G6‐treated mice did not differ (Figure S4). Fasting serum TG levels were significantly reduced in Ad‐shPLA2G6‐treated HFD‐fed mice compared to Ad‐shLuc‐treated HFD‐fed mice (Figure 3E). In contrast, the levels of serum FFA, total cholesterol, and HDL cholesterol did not differ significantly between Ad‐shLuc‐ and Ad‐shPLA2G6‐treated mice (Figure 3F–H). These results indicate that liver‐specific *Pla2g6* knockdown attenuates hepatic lipid accumulation and serum TG levels without affecting adipose morphology or serum FFAs.

### 3.4 Suppression of PLA2G6 in the Liver Improves Glucose Metabolism

**FIGURE 4:**
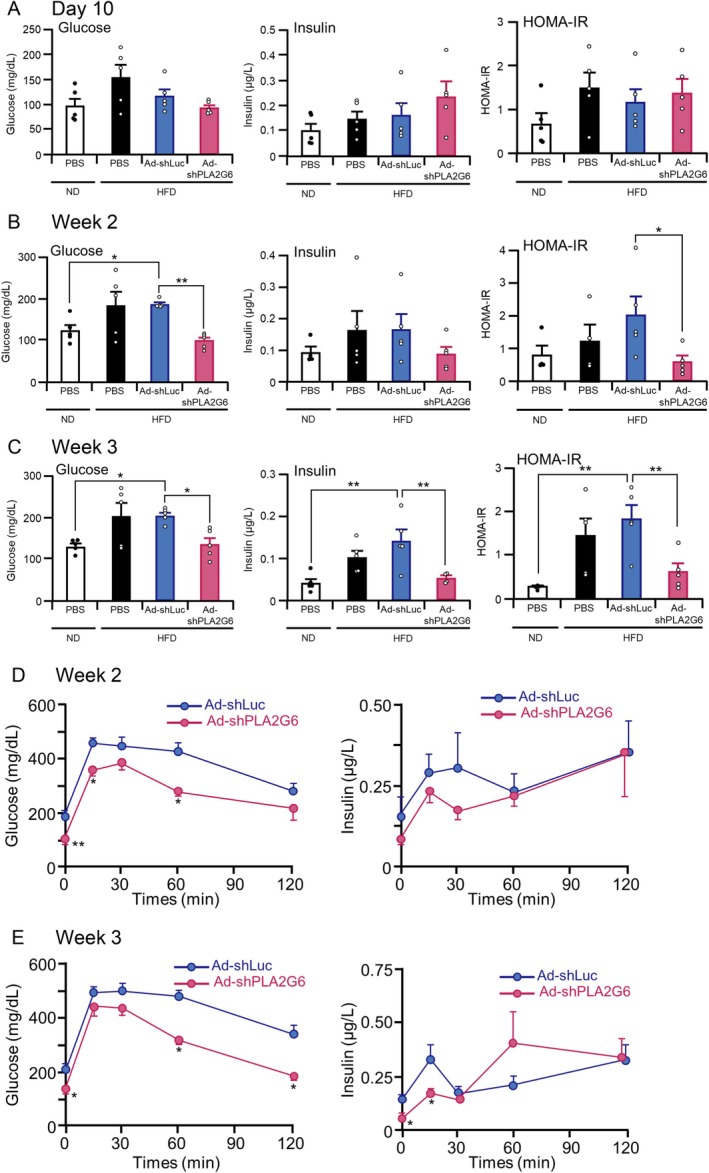
Suppression of *Pla2g6* in the liver improves glucose metabolism. (A–C) Fasting blood glucose levels, fasting insulin levels, and homeostasis model assessment of insulin resistance (HOMA‐IR) in normal diet (ND)‐ or high‐fat diet (HFD)‐fed C57BL/6 mice at (A) 10 days, (B) 2 weeks, and (C) 3 weeks after administration of Ad‐shPLA2G6, Ad‐shLuc, or PBS. (D, E) Blood glucose and insulin levels during the intraperitoneal glucose tolerance test of HFD‐fed C57BL/6 mice at weeks (D) 2 or (E) 3 after administration of Ad‐shPLA2G6 or Ad‐shLuc. Data are expressed as mean ± SE (*n* = 4–5). *p < 0.05 and **p < 0.01 assessed using one‐way ANOVA with Dunnett's post hoc test (for multiple comparisons) (A–C) or Mann–Whitney *U* test (for two independent group comparisons) (D, E).

To evaluate the effect of hepatic suppression of *Pla2g6* on glucose metabolism, we monitored blood glucose and insulin levels under fasting conditions 10 days after the administration of Ad‐shPLA2G6, Ad‐shLuc, or PBS. No significant differences in blood glucose, insulin levels, and HOMA‐IR were observed between Ad‐shPLA2G6‐ and Ad‐shLuc‐treated HFD‐fed mice (Figure 4A). However, 2 weeks after the administration of Ad vectors, fasting blood glucose levels were significantly reduced in Ad‐shPLA2G6‐treated HFD‐fed mice than in Ad‐shLuc‐treated HFD‐fed mice and were similar to those in PBS‐treated ND‐fed mice (Figure 4B). The fasting insulin levels tended to be lower in Ad‐shPLA2G6‐treated HFD‐fed mice than in Ad‐shLuc‐treated HFD‐fed mice (Figure 4B). However, HOMA‐IR was significantly lower in Ad‐shPLA2G6‐treated mice than in Ad‐shLuc‐treated mice and was comparable to that in PBS‐treated ND‐fed mice (Figure 4B). At week 3 after the treatments, fasting glucose, insulin, and HOMA‐IR levels in HFD‐fed mice were significantly lower in Ad‐shPLA2G6‐treated mice than in Ad‐shLuc‐treated mice (Figure 4C). Furthermore, intraperitoneal glucose tolerance tests at 2 and 3 weeks after Ad vector administration indicated improved glucose tolerance in Ad‐shPLA2G6‐treated HFD‐fed mice, compared with their Ad‐shLuc‐treated counterparts (Figure 4D,E). These results indicate that *Pla2g6* suppression in the liver improves insulin tolerance and glucose metabolism.

### 3.5 Suppression of PLA2G6 in the Liver Is Associated With Lipid Metabolism

**FIGURE 5:**
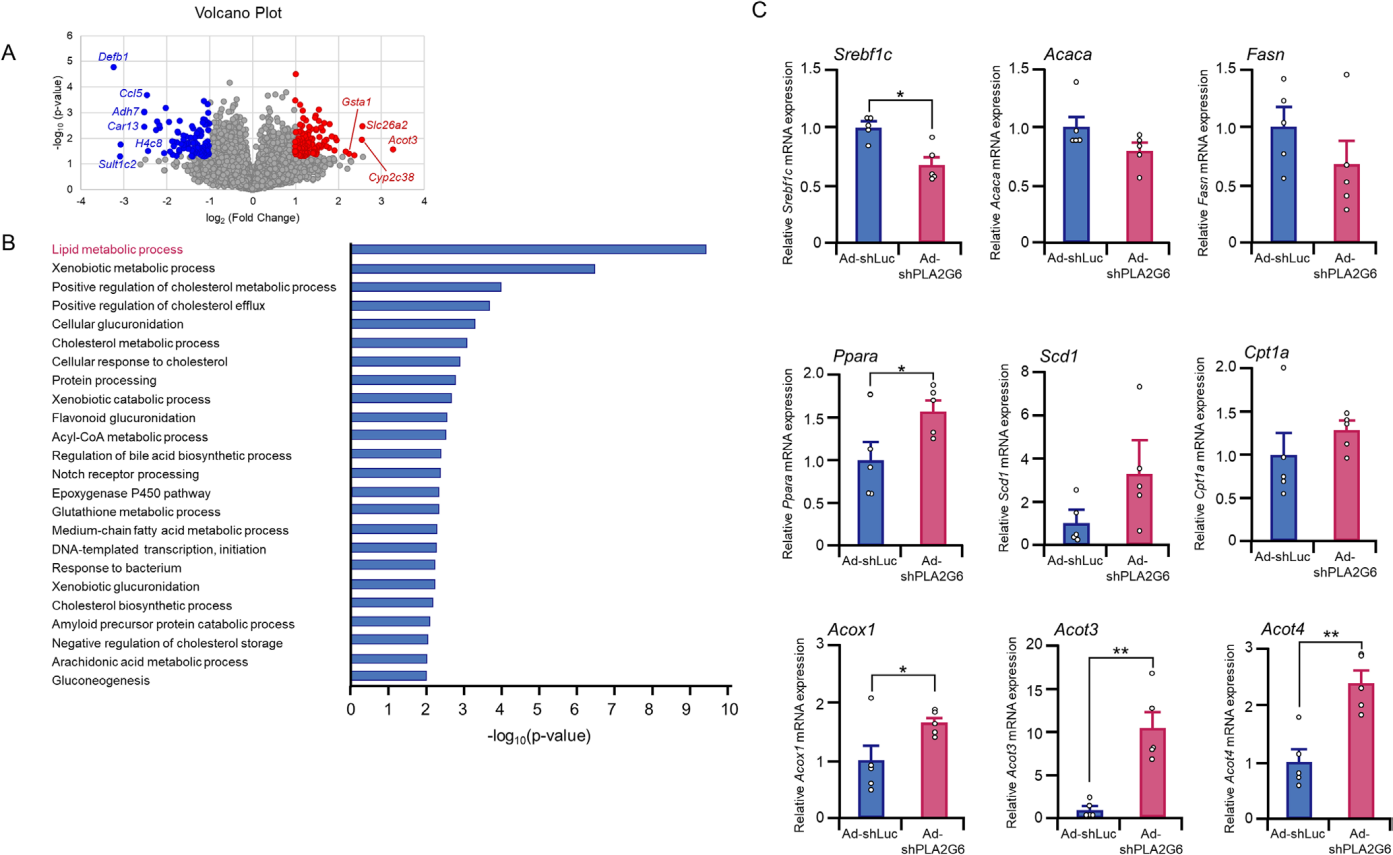
Hepatic *Pla2g6* inhibition alters the expression of lipid metabolism‐related genes. C57BL/6 mice on a high‐fat diet (HFD) received intravenous tail vein injections of Ad‐shPLA2G6 or Ad‐shLuc. Ten days after treatment initiation, livers were collected for RNA extraction and analysis. (A) Volcano plot of the differentially expressed genes identified using RNA‐seq analysis. Each point represents a single gene. Significantly upregulated genes (log_2_[fold‐change] > 1 and *p*‐value < 0.05) are indicated in red, and significantly downregulated genes (log_2_[fold‐change] < −1 and *p*‐value < 0.05) are indicated in blue. Non‐significant genes are indicated in gray. The top 10 differentially expressed genes are annotated on the volcano plot. (B) Top 24 enriched GO (Biological Process) terms of differentially expressed genes. Differentially expressed genes between Ad‐shPLA2G6‐ and Ad‐shLuc‐treated C57BL/6 mouse liver were subjected to GO enrichment analysis. The *X*‐axis indicates –log_10_ (*p*‐value), and the *Y*‐axis lists the top GO terms. (C) Expression levels of lipid metabolism‐related genes in the livers of mice at 10 days after the administration of Ad‐shPLA2G6 or Ad‐shLuc. Data are expressed as mean ± SE (*n* = 5). *p < 0.05, **p < 0.01, compared to Ad‐shLuc treatment assessed using the Mann–Whitney *U* test.

To explore the molecular mechanisms underlying the effects of hepatic suppression of *Pla2g6* on glucose and lipid metabolism, we performed RNA sequencing (RNA‐seq) analysis using liver samples from Ad‐shPLA2G6 and Ad‐shLuc‐treated mice. Differential gene expression analysis identified 119 upregulated and 104 downregulated genes in Ad‐shPLA2G6‐treated mice compared with those in Ad‐shLuc‐treated mice (adjusted *p* < 0.05, |log_2_ fold change| > 1) (Figure 5A). GO analysis revealed that differentially expressed genes in the “lipid metabolic processes” category included *Acot3*, *Acot4*, *Cyp2C38*, *Adh7*, and *Pla2g6* (Figure 5B). Therefore, we focused on the lipid synthesis‐ and degradation‐related genes in the liver. The mRNA level of *Srebf1c*, a master regulator of lipid metabolism, was 1.5‐fold lower in Ad‐shPLA2G6‐treated mouse livers than in Ad‐shLuc‐treated mouse livers (Figure 5C). Consistent with reduced hepatic *Srebf1c* expression, the mRNA levels of key de novo lipogenesis genes, *Fasn* and *Acaca*, tended to be lower in the livers of Ad‐shPLA2G6‐treated mice than in those of Ad‐shLuc‐treated mice. Furthermore, evaluation of the expression of fatty acid oxidation‐related genes indicated 1.6‐fold higher expression of *Pparα* mRNA in Ad‐shPLA2G6‐treated mouse livers than in Ad‐shLuc‐treated mouse livers (Figure 5C). Hepatic *Scd1* mRNA levels in Ad‐shPLA2G6‐treated mice tended to be higher than those in Ad‐shLuc‐treated mice. Fatty acid oxidation occurs in the mitochondria and peroxisomes [41]. Therefore, we assessed the expression of CPT1A, a mitochondrial fatty acid oxidation enzyme. No significant differences in *Cpt1a* mRNA levels were observed between Ad‐shPLA2G6‐ and Ad‐shLuc‐treated mouse livers (Figure 5C). Additionally, the mRNA levels of mitochondrial genes, including *Ndufab1* and *Cpt2*, did not differ significantly between Ad‐shPLA2G6‐ and Ad‐shLuc‐treated mice (Figure S5). Furthermore, the hepatic mRNA levels of *Acox1*, a peroxisomal fatty acid oxidation enzyme [42], were 1.7‐fold higher in Ad‐shPLA2G6‐treated mice than those in Ad‐shLuc‐treated mice (Figure 5C). The expression of peroxisomal genes involved in fatty acid oxidation, including *Acot3* and *Acot4*, was 10.4‐ and 2.4‐fold higher, respectively, in Ad‐shPLA2G6‐treated mouse livers than in Ad‐shLuc‐treated mouse livers. No significant differences in *Srebf1c*, *Pparα*, *Cpt1a*, *Acox1*, *Acot3*, and *Acot4* expression levels were observed between DMSO‐ and FKGK18‐treated mouse livers (Figure S6). These findings suggest that hepatic PLA2G6 suppression reduces lipid synthesis and upregulates fatty acid oxidation in peroxisomes.

## 4 Discussion

Hepatic lipid accumulation is closely linked to insulin resistance, contributing to impaired regulation of glucose and lipid metabolism in T2DM [43, 44]. In this study, we demonstrated that suppression of *Pla2g6*, either pharmacologically with FKGK18 or via liver‐specific knockdown using Ad‐shPLA2G6, reduced hepatic lipid accumulation in HFD‐fed mice. In particular, the liver‐specific suppression of PLA2G6 reduced serum TG, fasting blood glucose, and insulin levels. Lipidomic analysis revealed that *Pla2g6* suppression altered hepatic phospholipids, including PC, LPC, and PE species, and fatty acid metabolites, suggesting that hepatic phospholipid remodeling plays a central role in protecting against HFD‐induced T2DM and MASLD.

The phenotype observed after pharmacological inhibition of PLA2G6 using FKGK18 differed from that observed in liver‐specific *Pla2g6* knockdown. FKGK18 treatment reduced hepatic lipid droplet size without affecting the serum TG and fasting blood glucose levels in HFD‐fed mice, whereas Ad‐shPLA2G6 reduced hepatic lipid accumulation as well as serum TG and fasting blood glucose levels. This discrepancy is likely attributable to the weaker suppression of hepatic PLA2G6 activity achieved by the inhibitor compared with liver‐specific *Pla2g6* knockdown. Although FKGK18 modestly reduced hepatic *Srebf1c* mRNA levels, Ad‐shPLA2G6 administration suppressed *Srebf1c* mRNA expression and upregulated fatty acid oxidation‐related genes. A higher dose or more frequent administration of FKGK18 may possibly yield effects comparable to those observed with liver‐specific PLA2G6 knockdown.

Excessive hepatic lipid accumulation impairs insulin signaling, promoting insulin resistance and diminishing suppression of gluconeogenesis that in turn contributes to elevated fasting blood glucose levels [45, 46]. In this study, Ad‐shPLA2G6‐mediated *Pla2g6* knockdown in the liver ameliorated HFD‐induced lipid accumulation and reduced serum TG levels. This effect was associated with decreased *Srebf1c* levels and increased expression of fatty acid oxidation‐related mRNAs, contributing to reduced lipid synthesis and enhanced lipid degradation. *Scd1* expression in Ad‐shPLA2G6‐mouse liver tended to be upregulated despite reduced *Srebf1c* expression, suggesting that *Scd1* may be regulated independently of *Srebf1c* and potentially via *Pparα* [47]. Although fatty acid oxidation occurs in mitochondria and peroxisomes, *Pla2g6* suppression reduced *Acox1* expression—the rate‐limiting enzyme in peroxisomal fatty acid oxidation. Very‐long‐chain fatty acids, oxidized exclusively in peroxisomes, are shortened to acyl‐CoAs (the activated form of FFAs) and shuttled to the mitochondria for complete oxidation. Activation of peroxisomal fatty acid oxidation reduces hepatic lipid accumulation, whereas enhanced mitochondrial fatty acid oxidation further suppresses gluconeogenesis and, via PPARα‐mediated pathways, promotes peripheral glucose utilization, thereby improving insulin sensitivity [48, 49]. These findings suggest that enhanced *Pparα*‐driven fatty acid oxidation, rather than reduced *Srebf1*‐mediated lipogenesis alone, may play a predominant role in the reduction of hepatic lipid accumulation under liver‐specific PLA2G6 suppression. Together, these alterations alleviate hepatic lipid accumulation and lower serum TG levels, leading to improvements in glucose tolerance, fasting blood glucose levels, and insulin sensitivity.

Liver‐specific *Pla2g6* suppression via Ad‐shPLA2G6 altered multiple phospholipid and fatty acid metabolite species. Compared with Ad‐shLuc‐treated mice, Ad‐shPLA2G6‐treated mice exhibited marked changes in PC 38:6, LPC 18:0, LPC 18:1, and various fatty acid metabolites. These findings are consistent with those of previous studies, indicating that *Pla2g6* knockout increases PC 38:6 and decreases LPC 18:0 and PGE_2_ [20, 21]. Furthermore, LPC 16:0 was significantly decreased in *Pla2g6* knockout mouse livers and tended to decrease in Ad‐shPLA2G6‐treated mice [20, 21]. Studies have demonstrated that PC 32:0 is decreased in PLA2G6‐transfected 293 cells [50] but increased in Ad‐shPLA2G6‐treated mouse livers. Taken together, these findings suggest that liver‐specific suppression of *Pla2g6* induces alterations in lipid profiles.

PLA2G6 deficiency ameliorates hepatic steatosis and reduces serum TG levels in diabetic ob/ob and HFD‐fed mice [20, 21]. Consistently, our results reveal liver‐specific suppression of *Pla2g6* via Ad‐shPLA2G6 attenuated hepatic lipid accumulation, lowered serum TG levels, and suppressed the increase in blood glucose levels in HFD‐fed mice. As described above, the changes in hepatic PC, LPC, and PGE_2_ profiles exhibited similar trends in *Pla2g6* knockout mice, suggesting that hepatic phospholipid remodeling plays a key role in protecting against hepatic lipid accumulation and impaired glucose metabolism under conditions of PLA2G6 deficiency and liver‐specific suppression.

In Ad‐shPLA2G6‐treated mice, the hepatic levels of LPC 18:1 increased. Previous studies have demonstrated that C18:0 (stearic acid), similar to C16:0 (palmitic acid), exhibit hepatic lipotoxicity and contribute to MASLD progression [51, 52]. Conversely, C18:1 (oleic acid), a monounsaturated fatty acid, has been shown to be associated with PPARα activation and improved insulin sensitivity [53, 54]. Furthermore, patients with MASLD typically exhibit higher levels of total saturated fatty acids, such as palmitic acid, than monounsaturated fatty acids, such as oleic acid [55]. In cellular models, palmitic acid inactivates PPARα [56], which plays an important role in controlling fatty acid oxidation, whereas oleic acid reduces palmitic acid‐mediated hepatocellular lipotoxicity [57]. Thus, an increase in oleic acid by the suppression of *Pla2g6* may contribute to the improvement of insulin resistance via activation of PPARα.

PLA2G6 suppression also increased hepatic PC 38:6, a DHA (C22:6)‐containing phospholipid, and decreased *Srebf1c* mRNA levels. DHA‐containing phospholipids inhibit lipogenesis via suppression of *Srebf1c* [58, 59], suggesting that elevated DHA‐PC could be the underlying mechanism for the observed reduction in hepatic lipid synthesis. Furthermore, 12‐HETE and PGE_2_ levels were markedly reduced in the Ad‐shPLA2G6‐treated mice. Previous reports have demonstrated that 12‐HETE, a metabolite of arachidonic acid hydrolyzed by PLA_2_ and oxidized by lipoxygenases [60], was increased in mice with metabolic dysfunction associated with steatohepatitis induced by methionine‐ and choline‐deficient diet treatment [61]. PGE_2_, a metabolite of arachidonic acid synthesized via the cyclooxygenase pathway, contributes to hepatic insulin resistance [62]. Reduction in 12‐HETE and PGE_2_ levels in the livers of Ad‐shPLA2G6 mice may lead to suppression of hepatic lipid accumulation and improvement of hepatic insulin resistance. Taken together, these findings suggest that changes in phospholipid and fatty acid metabolite profiles resulting from liver‐specific Pla2g6 suppression may improve glucose and lipid metabolism. However, further mechanistic studies are needed to clarify the causal relationships among these factors.

Several studies have demonstrated that PLA2G6 plays a protective role in various biological processes, including neuronal function [18], sperm fertility [17], and insulin secretion [19]. Given its ubiquitous expression, PLA2G6 likely contributes to maintaining physiological functions across multiple organs. In contrast, PLA2G6 expression levels were increased in the livers of HFD‐fed C57BL/6 mice. Therefore, the improvements in glucose and lipid metabolism observed in this study are likely attributable to the suppression of excessive PLA2G6 activity.

Our findings suggest that targeting hepatic Pla2g6 modulates phospholipid and fatty acid metabolites, thereby improving glucose and lipid homeostasis. One limitation of the present study is that the precise molecular mechanisms linking liver‐specific PLA2G6 suppression to reduced hepatic lipid accumulation and improved insulin sensitivity were not directly examined. Further mechanistic studies are required to determine which specific lipid alterations causally contribute to these metabolic improvements. Additionally, this study was conducted in HFD‐fed mouse models, which may not fully recapitulate the complexity of human pathophysiology. Although our results provide insights into potential mechanisms, their translational relevance to humans remains to be established. Future investigations using human tissues or clinical samples will be necessary to validate and extend our findings.

In summary, liver‐specific suppression of PLA2G6 attenuated hepatic lipid accumulation, serum TG levels, and fasting blood glucose levels by altering the hepatic phospholipid and fatty acid metabolite profiles. These findings suggest PLA2G6 as a potential therapeutic target for the treatment of MASLD and T2DM.

## Author Contributions

K.S. conceived and designed the project, performed the experiments, analyzed the data, and wrote the manuscript. M.N., S.M., and M.K. performed the experiments. K.N. performed and analyzed the lipid measurements. S.H. analyzed the images. M.H.‐I., F.S., and T.N. provided the materials. H.S. analyzed the lipid measurements and revised the manuscript. K.T. analyzed the data and revised the manuscript. H.M. provided materials and supervised the project. All authors have approved the manuscript.

## Funding

This work was supported by Japan Society for the Promotion of Science (JSPS) KAKENHI (JP21K06680, JP25K03046, JP23H00552), Mishima Kaiun Memorial Foundation, Mishima Kaiun Memorial Foundation, BOMU MELLNESS FOUNDATION, Takara Bio Research Award, Osaka Ohtani University Research Foundation, Japan Agency for Medical Research and Development (AMED), 25fk0210150, NCGM Intramural Research Fund (22T001, 21A2006, 24A2011), Platform Project for Supporting Drug Discovery and Life Science Research (Basis for Supporting Innovative Drug Discovery and Life Science Research [BINDS]) from AMED (JP25ama121052), Foundation of Kinoshita Memorial Enterprise.

## Ethics Statement

All animal procedures were approved by the Institutional Animal Care and Use Committee of Osaka Ohtani University (approval IDs 1407 and 2002) and The University of Osaka (approval ID doyaku R06‐3), and were performed in accordance with the Institutional Guidelines and Regulations for Animal Experiments at Osaka Ohtani University and The University of Osaka.

## Conflicts of Interest

The authors declare no conflicts of interest.

## Supporting information


**Figure S1:** fsb271821‐sup‐0001‐FigureS1.pdf. *Pla2g6* mRNA expression in the hepatocytes and nonparenchymal cells. *Pla2g6* mRNA levels of the hepatocytes (A) or nonparenchymal cells (B) in C57BL/6 mice fed an ND or HFD for 4 weeks, starting at 6 weeks of age. *Pla2g6* mRNA levels in ND‐fed mice were set to 1.0. ND, normal diet; HFD, high‐fat diet.
**Figure S2:** Hepatic *Pla2g6* mRNA expression in mice 8 weeks after administration of Ad vector. Hepatic *Pla2g6* mRNA levels at 8 weeks after the administration of Ad‐shPLA2G6, Ad‐shLuc, or PBS. *Pla2g6* mRNA levels in Ad‐shLuc‐treated mice were set to 1.0. One‐way ANOVA with Dunnett's post hoc test was used for multiple comparisons. The data are expressed as mean ± SE values (*n* = 4–5). ND, normal diet; HFD, high‐fat diet. **p < 0.01.
**Figure S3:** Intravenous administration of Ad‐shPLA2G6 did not affect PLA2G6 expression in adipose tissue. *Pla2g6* mRNA levels in the adipose tissue of HFD‐fed C57BL/6 mice at 10 days after the administration of Ad‐shPLA2G6, Ad‐shLuc, or PBS, as determined using quantitative reverse transcription‐polymerase chain reaction. The mRNA levels in Ad‐shLuc‐treated mice were set to 1.0. Data are expressed as mean ± SE (*n* = 5). One‐way ANOVA with Dunnett's post hoc test was used for multiple comparisons.
**Figure S4:** Hematoxylin and eosin staining of adipose tissues of Ad‐shPLA2G6‐treated mice. Hematoxylin and eosin staining of epididymal adipose tissues of HFD‐fed C57BL/6 mice 10 days after administration of Ad‐shPLA2G6, Ad‐shLuc, or PBS. Scale bar = 50 μm.
**Figure S5:** Suppression of *Pla2g6* in the liver does not affect mitochondrial gene expression in the liver. Mitochondrial gene expression in the livers of HFD‐fed C57BL/6 mice at 10 days after the administration of Ad‐shPLA2G6 or Ad‐shLuc as determined using quantitative reverse transcription‐polymerase chain reaction. The mRNA levels in Ad‐shLuc‐treated mice were set to 1.0. Data are expressed as mean ± SE (*n* = 5). Statistical analysis was performed using the Mann–Whitney *U* test to compare the differences between the two independent groups.
**Figure S6:** Intraperitoneal administration of the PLA2G6 inhibitor did not affect lipid metabolism‐related gene expression in the liver. Lipid metabolism‐related gene expression in the livers of HFD‐fed C57BL/6 mice at 10 days after the initiation of FKGK18 (PLA2G6 inhibitor) or DMSO treatment. Data are expressed as mean ± SE (*n* = 5). The Mann–Whitney *U* test was used to compare the differences between the two independent groups.

**Table S1:** Oligonucleotide sequences used for shRNA.
**Table S2:** Primer sequences used for quantitative RT‐PCR.
**Table S3:** Changes in liver fatty acid metabolites 10 days after administration of Ad vectors.

## Data Availability

The Gene Expression Omnibus (GEO) accession number for the RNA‐sequencing analysis is GSE310622. RNA‐seq data have been deposited at GEO and are publicly available as of the date of publication.
