# Supplementary figures and images for: Liver‐Specific Suppression of PLA2G6/iPLA2β Improves Glucose and Lipid Metabolism in High‐Fat Diet‐Fed Mice
Source: FASEB J. 2026 Apr 20;40(8):e71821. doi: 10.1096/fj.202504753RR (PMC13094463; doi:10.1096/fj.202504753RR)

Figure S1

A

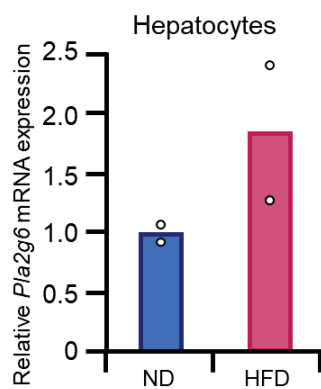

B

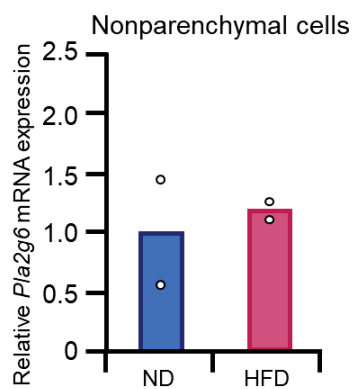

Figure S2

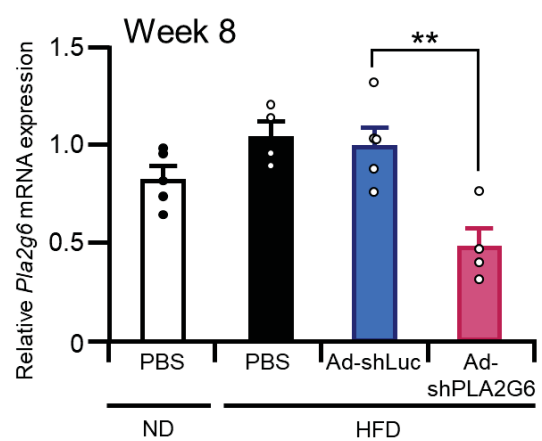

Figure S3

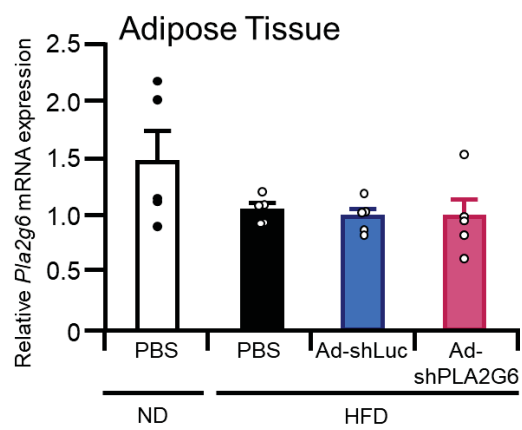

Figure S4

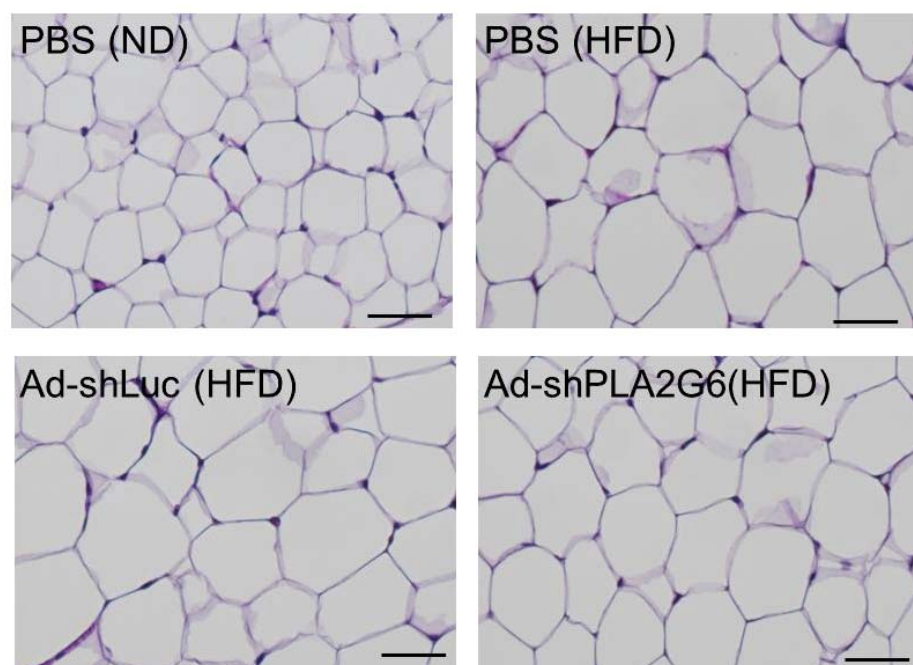

Figure S5

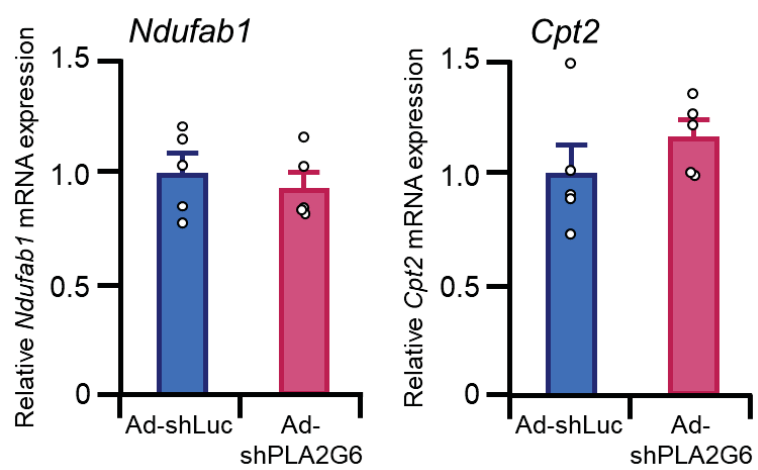

Figure S6

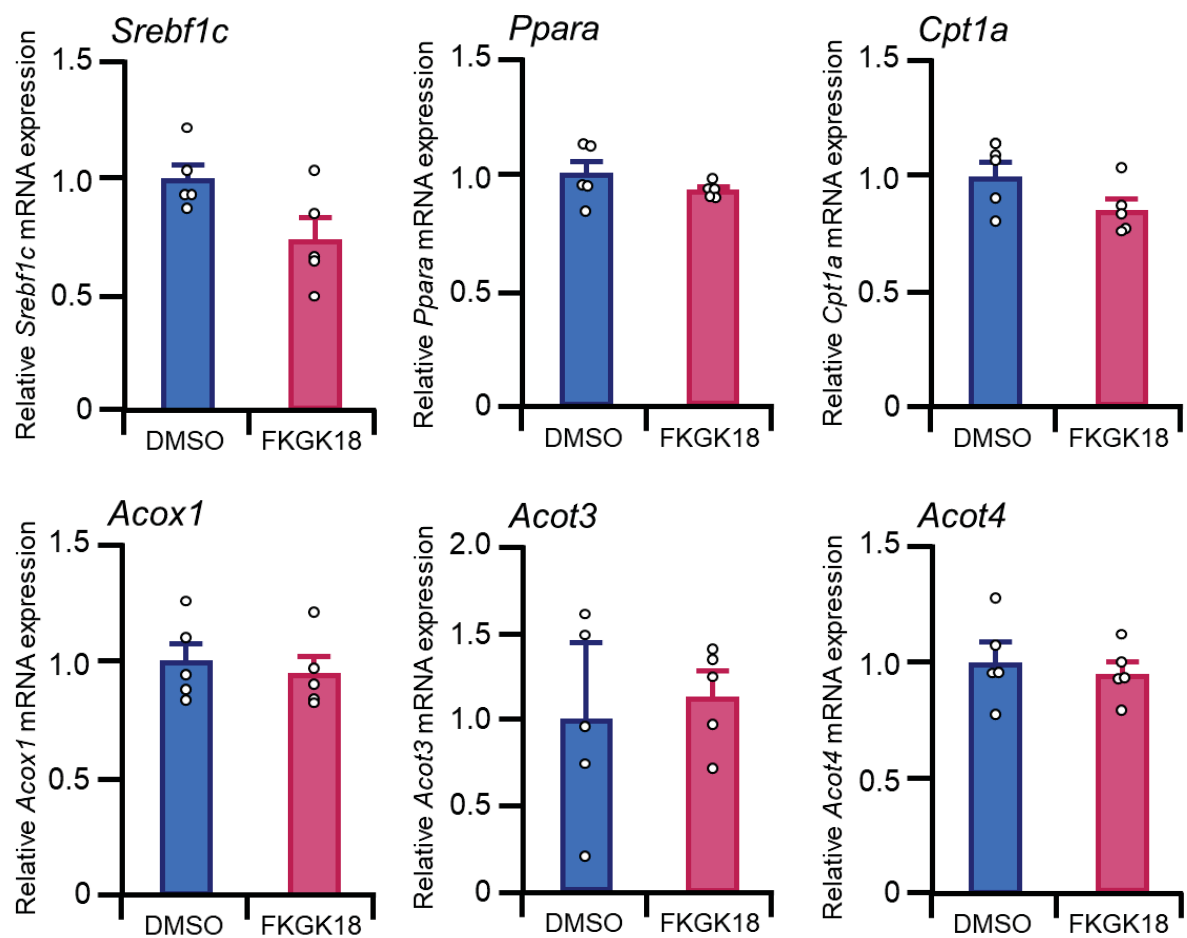

Supplement: Supplementary file 1 — Figure S1: fsb271821‐sup‐0001‐FigureS1.pdf. Pla2g6 mRNA expression in the hepatocytes and nonparenchymal cells. Pla2g6 mRNA levels of the hepatocytes (A) or nonparenchymal cells (B) in C57BL/6 mice fed an ND or HFD for 4 weeks, starting at 6 weeks of age. Pla2g6 mRNA levels in ND‐fed mice were set to 1.0. ND, normal diet; HFD, high‐fat diet. Figure S2: Hepatic Pla2g6 mRNA expression in mice 8 weeks after administration of Ad vector. Hepatic Pla2g6 mRNA levels at 8 weeks after the administration of Ad‐shPLA2G6, Ad‐shLuc, or PBS. Pla2g6 mRNA levels in Ad‐shLuc‐treated mice were set to 1.0. One‐way ANOVA with Dunnett's post hoc test was used for multiple comparisons. The data are expressed as mean ± SE values (n = 4–5). ND, normal diet; HFD, high‐fat diet. **p < 0.01. Figure S3: Intravenous administration of Ad‐shPLA2G6 did not affect PLA2G6 expression in adipose tissue. Pla2g6 mRNA levels in the adipose tissue of HFD‐fed C57BL/6 mice at 10 days after the administration of Ad‐shPLA2G6, Ad‐shLuc, or PBS, as determined using quantitative reverse transcription‐polymerase chain reaction. The mRNA levels in Ad‐shLuc‐treated mice were set to 1.0. Data are expressed as mean ± SE (n = 5). One‐way ANOVA with Dunnett's post hoc test was used for multiple comparisons. Figure S4: Hematoxylin and eosin staining of adipose tissues of Ad‐shPLA2G6‐treated mice. Hematoxylin and eosin staining of epididymal adipose tissues of HFD‐fed C57BL/6 mice 10 days after administration of Ad‐shPLA2G6, Ad‐shLuc, or PBS. Scale bar = 50 μm. Figure S5: Suppression of Pla2g6 in the liver does not affect mitochondrial gene expression in the liver. Mitochondrial gene expression in the livers of HFD‐fed C57BL/6 mice at 10 days after the administration of Ad‐shPLA2G6 or Ad‐shLuc as determined using quantitative reverse transcription‐polymerase chain reaction. The mRNA levels in Ad‐shLuc‐treated mice were set to 1.0. Data are expressed as mean ± SE (n = 5). Statistical analysis was performed using the Mann– [file FSB2-40-e71821-s002.pdf]
