# Supplementary material for: Liver‐Specific Suppression of PLA2G6/iPLA2β Improves Glucose and Lipid Metabolism in High‐Fat Diet‐Fed Mice
Source: FASEB J. 2026 Apr 20;40(8):e71821. doi: 10.1096/fj.202504753RR (PMC13094463; doi:10.1096/fj.202504753RR)
Supplement: Supplementary file 2 — Table S1: Oligonucleotide sequences used for shRNA. Table S2: Primer sequences used for quantitative RT‐PCR. Table S3: Changes in liver fatty acid metabolites 10 days after administration of Ad vectors. [file FSB2-40-e71821-s001.docx]

**Table S1.** Oligonucleotide sequences used for shRNA

| **Gene** | **Sequences (5'–3')** |
| --- | --- |
| shPLA2G6 sense | CCGGTCAAAGCCCTCATTGTATTCTGTGAAGCCACAGATGGGAATACAATGAGGGCTTTGACCTTTTTTG |
| shPLA2G6 antisense | TCGACAAAAAAGGTCAAAGCCCTCATTGTATTCCCATCTGTGGCTTCACAGAATACAATGAGGGCTTTGACCGG |
| shLuc sense | CCACGCTGAGTACTTCGAAATTTCAAGAGAATTTCGAAGTACTCAGCGTTTTTTGGAAAT |
| shLuc antisense | TCGAATTTCCAAAAAACGCTGAGTACTTCGAAATTCTCTTGAAATTTCGAAGTACTCAGCGTGG |

Target sequences for the gene are underlined.

**Table S2.** Primer sequences used for quantitative RT-PCR

| **Gene** | **Forward primer (5'–3')** | **Reverse primer (5'–3')** |
| --- | --- | --- |
| *Pla2g6* | CGGCCTGAACCAGGTAAACAA | GTTGCAGCGGGCATTACAG |
| *Srebf1* | TGACCCGGCTATTCCGTGA | CTGGGCTGAGCAATACAGTTC |
| *Ppara* | AACATCGAGTGTCGAATATGTGG | CCGAATAGTTCGCCGAAAGAA |
| *Acaca* | CGCCAACAATGGTATTGCAGC | TCGGATTGCACGTTCATTTCG |
| *Fasn* | AGAGATCCCGAGACGCTTCT | GCTTGGTCCTTTGAAGTCGAAGA |
| *Scd1* | TTCTTGCGATACACTCTGGTGC | CGGGATTGAATGTTCTTGTCGT |
| *Cpt1a* | CTCCGCCTGAGCCATGAAG | CACCAGTGATGATGCCATTCT |
| *Ndufab1* | TTTGTGCCGCCAGTACAGTG | TCAAACCCAAATTCGTCTTCCA |
| *Cpt2* | CAAAAGACTCATCCGCTTTGTTC | CATCACGACTGGGTTTGGGTA |
| *Acox1* | ATGGGTCATGGAACTCATCT | ACCACTTGATGGAAGTCACA |
| *Acot3* | CTATATCCAACATCGGCGGAAA | TCTTGACTCGCTTGGTGTTTATG |
| *Acot4* | AGCAGTGCGGTACATGCTTC | AGAGCCATTGATGGAAACTGTG |
| *Actb* | GGCTGTATTCCCCTCCATCG | CCAGTTGGTAACAATGCCATGT |

**Table S3.** Changes in liver fatty acid metabolites 10 days after administration of Ad vectors

|  | **Fatty acid metabolites** | **Ad-shLuc**  **(pg/mg)** | **Ad-shPLA2G6 (pg/mg)** |
| --- | --- | --- | --- |
| **Arachidonic-derived fatty acid metabolites** | 11-HETE | 2.45±0.04 | 1.94±0.27 |
|  | 11,12-DHET | 2.36±0.20 | 3.19±0.26 |
|  | 12-HETE | 3.11±0.32 | 1.49±0.14 |
|  | 14,15-DHET | 7.88±0.74 | 12.02±1.00 |
|  | 18-HETE | 1.19±0.21 | 2.87±0.77 |
|  | 5,6-DHET | 0.14±0.03 | 0.28±0.03 |
|  | 8,9-DHET | 0.54±0.10 | 1.07±0.17 |
|  | PGE_2_ | 0.21±0.05 | 0.08±0.01 |
|  | PGF_2α_ | 0.47±0.12 | 0.18±0.04 |
| **DHA-derived**  **fatty acid metabolites** | 10-HDHA | 2.09±0.45 | 0.93±0.31 |
|  | 16-HDHA | 1.97±0.40 | 0.68±0.08 |
|  | 8-HDHA | 2.70±0.30 | 1.43±0.33 |

Fatty acid metabolites with significant differences among the 51 fatty acid metabolites were measured. The data are expressed as mean ± SE values (n = 4–5).
